# Demystifying impact evaluation: an impact evaluation framework

**DOI:** 10.3389/fepid.2025.1460997

**Published:** 2025-03-18

**Authors:** Janet Michel, Kimon Schneider

**Affiliations:** ^1^ Swiss Tropical and Public Health Institute (Swiss TPH), Basel, Switzerland; ^2^ Swiss Centre for International Health (SCIH), University of Basel, Basel, Switzerland; ^3^ ksc Consultancy, Zurich, Switzerland

**Keywords:** impact evaluation, attribution, contribution, theory of change, baseline data, midline data, endline data, context

## Abstract

As the global financial, economic, social, environmental, political, technological and health crises deepen and become more complex, funders are increasingly eliciting for programs/research that demonstrate impact. A lot of evaluations often lack the methodological robustness to inform further action by failing to demonstrate the context mechanism and outcome pathways. The landscape is changing. The value of programs/interventions and research is increasingly coming under scrutiny. Impact evaluation is the process of determining to what extent observed changes in the outcome are attributable to the intervention. Figures alone cannot explain why things are that way, and stories alone cannot demonstrate who or how many people benefited and to what extent. Additional methodological tools, such as participatory methods, theories of change, and human centred designs citizen science and the engagement of all key stakeholders, including those previously known as beneficiaries is fundamental. This facilitates a better understanding of the problems while unraveling potential solutions, bearing in mind that any health system intervention can have positive, negative, intended, unintended, direct and indirect consequences. Transdisciplinary, multi and inter-disciplinary approaches and mixed methods therefore become indispensable. To that end we propose an impact evaluation framework with seven central tenets namely; Theory of change (TOC) or program theory, Stakeholder engagement including beneficiaries, Use of mixed method indicators, Baseline of outcome of interest, Midline assessment of outcome of interest, Endline assessment of outcome of interest and Validation/Co-creation.

## Background

Research over the past few decades has grown, outstripping available public funding in many countries. This has led to discussions about how to get the best value from research or interventions. A growing interest is the non-academic benefits or impact of research as funders and Governments world-wide, seek evidence of the value of research investments to society (1). The financial crisis, dwindling resources at the backdrop of multiple global health, environmental, economic, political, security and climate change crises have exacerbated the need to demonstrate impact. Complex problems caused by a confluence of the above-mentioned factors accompanied by conflict, pandemics, massive worker resignations and search for meaning, call for new ways of monitoring and evaluation with the methodological robustness to inform further action. In addition, many communities are exhibiting research fatigue and are increasingly demanding to see the benefits of years of research or interventions being rolled out in their communities (2, 3). Most research or interventions need resources and all research or interventions can have both intended and unintended effects. The world crises make funding for research and interventions scarce and highly competitive. Those who receive funding are increasingly facing demands to demonstrate impact. Impact evaluation is the process of determining to what extent observed changes in the outcome are attributable to the intervention, both the intended and unintended consequences (4). In research, impact is defined as a positive effect on, change, or benefit to society, culture, the environment, or the economy and quality of life beyond the research environment/academia (5). The United Kingdom (UK) started to evaluate societal impact of research (the impact agenda) in 2014, with the aim to articulate the benefits and contribution of research to the health and well-being of the society, guided by the UK Research Excellence Framework (5).

### Black box evaluation

Impact evaluation to date has been limited to experimental and quasi-experimental designs. Not all phenomena can be studied through the above-mentioned designs. In fact, most research and interventions are observational in nature. Everyone wants to know that the work they do has value. Researchers and program managers often find themselves confronted by the need to demonstrate impact. Unprepared and not planned for, at design stage, it is difficult to demonstrate impact.

This has generated a resistance to impact evaluation with many researchers arguing that impact is difficult to demonstrate. Many interventions and programs have no theory of change (ToC) or program theory. Confronted by the need to demonstrate impact without a TOC or program theory, inevitably leads to black box impact evaluation (4). Blackbox evaluation is defined as producing evidence of the effect of a program, research without examining the elements of the research or program to determine how and why this led to the outcome (6). The landscape has and is changing. Despite increasing complexity, methodological challenges, and critical voices saying impact can't be measured, there is “hope” in the sense that there are newer, promising and pragmatic approaches out there, growing in number. The purpose of this view point is to demystify impact evaluation by proposing an impact evaluation framework with seven central tenets that enables researchers and program managers to design programs that capture impact or lack thereof as they unravel.

### Purposes of impact evaluation

An impact evaluation serves multiple purposes.

• as an accountability tool to funders (upward), beneficiaries (downward) and to self and team mates(horizontal) –something has been achieved.
• advocacy tool - to advocate with funders, government, the public, or others
• capture lessons learned
• analysis tool -to answer questions about program design, which bits work and which bits do not in what contexts and why?
• allocation tool - for allocating resources, possibly allocating more to those who have more impact
• ethical tool as it facilitates the capturing of intended and unintended effects thereby by preventing harm
• to promote evidence-informed decision-making in programming and research

## Imbedding impact aware methods at design stage

As stated above, demonstrating impact is not only restricted to experimental or quasi-experimental designs. Embedding impact-aware methods at program or research design stage is an ethical thing to do. All programs/research interventions need money, time and human and material resources. All interventions can have positive, negative, intended, unintended, direct and indirect consequences. Unfortunately, most M&E plans remain paper exercises. Many logic models - having the purpose to depict coherent results chains have been overly been overly misinterpreted with a focus on activities and outputs for example; 120 Nurses trained and 2 health facilities built with little or no focus on now that the health facilities have been built and the nurses trained, are the community members receiving the services they need, how is the quality and is the community now healthier? Another drawback of the overly activity and output-oriented logic models is the focus on quantitative indicators. This is because figures alone cannot explain why things are the way they are. Data and data sources, and perspectives triangulation are paramount in impact evaluation. Both quantitative and qualitative data are needed since stories alone cannot demonstrate who or how many people benefited, and to what extent. Methodological tools, such as participatory methods and human centered designs, support the engagement and participation of all key stakeholders, including beneficiaries, facilitating a better understanding of the problems and potential solutions, bearing in mind that any intervention can have positive, negative, intended, unintended effects/consequences.

Impact evaluation calls for the engagement of stakeholders affected or those with potential to affect the program. Beneficiaries are engaged with from the design stage through to evaluation, making impact evaluation not only easier but also possible., since the process also entails asking the beneficiaries the changes brought about into their lives as a result of the intervention.

## Demystifying impact evaluation

To demystify impact, we propose and define the following terms;

• Impact thinking- the thought process and action taken before intervention or research planning that ensures that impact is planned for.
• Impact aware methods- methods that include ToC, stakeholder and beneficiary involvement, use of empowering participatory methods like appreciative inquiry and reflective inquiry, collection of qualitative and quantitative indicators of outcome at baseline, midline and endline for a trend analysis to facilitate the demonstration of impact
• Validation/Co-creation
• Impact demonstration- data and data source triangulated results that show the pathways of contribution to change, how the intervention or research led to the outcomes.

Impact thinking before the research or intervention roll-out facilitates the embedding of impact aware methods at design stage, leading to the demonstration of impact or lack thereof as the program evolves rather than wait till the end for an external evaluator.

## Steps to demystifying impact-impact evaluation framework

1. Key stakeholder engagement

The process begins with the identification of the problem, the research or intervention to address the problem, who is affected by it and who influences the issue. The process is followed by a stakeholder map indicating positions and power of key stakeholders e.g., partners, stakeholders to engage with and stakeholders to consult.

This tool is to be used through out to answer the following questions.

• Who can affect or is affected by the intervention/research?
• In what way?
• What do they see as the problem
• What do they see as a solution?
• Do they see the research/intervention as contributing to the solution?
• How and in what way?
• Are they part of the process?

In order to speak of impact, the voice of the beneficiaries is key. This in not tantamount to a picture of one person saying, I benefitted but entails rigorous qualitative approaches that are participatory in nature (consensus) for example Realist Evaluation and Most significant change triangulated by quantitative indicators. Driven by stakeholder participation, equity, sustainability and the ethical use of evidence, impact evaluation provides decision-makers and stake-holders with comprehensive information about the consequences of the health interventions, policies, and projects (1).

2. Problem identification and articulation of a theory of change

As stated earlier, many interventions and programs have no ToC or program theory. ToC is defined as a comprehensive description, a statement that describes how and why change is expected to happen in a particular context. A ToC fills in the missing middle between intervention activities and desired goal by first identifying the desired long term goal, then working backwards to identify outcomes that ought to be in place first, the order and causal relationships leading to the goal (7). A theory of change is further broken down into program theory, a causal depiction of the causal chain from inputs to impact. This is expressed as a log frame with more explicit analysis of assumptions underlying theory. An intervention with a program theory allows for the testing of the validity of assumptions and the various links in the chain, using a variety of methods, and the building up of an argument as to whether the theory has been realized in practice.

**Figure 1:**
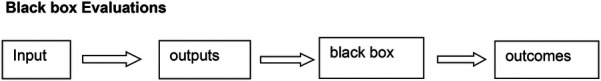
Black box evaluation.

As alluded to earlier, black box evaluations give a finding on impact with no indication as to how and why the intervention led to the outcome. Answering the why question requires looking inside the box, or along the results chain (4). The involvement of the key stakeholders, particularly beneficiaries identified above plays a critical role (Figure 1).

3. Baseline measurement of outcome of interest

Since impact evaluation is the process of determining to what extent observed changes in the outcome are attributed to the intervention, it is essential to;

• Define the starting point
• Define the outcome of interest if attribution is to be claimed.
• Carry out baseline measures of outcome of interest and capture both quantitative and qualitative indicators (narratives and stories)

4. Mid-point measure of outcome of interest

It is essential to track progress once the intervention roll-out or research has begun. The log frame is an important tool to monitor if progress is being made. The involvement of the key stakeholders ensures that deviations or detours or delays are identified early.

While as it might be too early to see a change in the outcome of interest, some short-term outcomes and outputs accompanied by narratives can give an indication of the direction the research or intervention is taking. Both quantitative and qualitative indicators measured at baseline are tracked and assessed at midline.

5. Endline measure of outcome of interest

Utilizing the log frame, end line indicators are quantitative and qualitative indicators tracked from baseline and at midline. The outcome of interest at baseline is measured at endline. The voices of the beneficiaries, key stakeholders are essential in describing if the destination has been reached (intended) or a wrong destination has been reached-unintended.

6. Mixed methods-use of qualitative and quantitative indicators to track outcome of interest

The quantitative and qualitative data complement each other and narratives are used to explain the figures. If discord is identified, additional data is required. This underscores the importance of mixed data collection and triangulation when demonstrating impact

7. Validation/co-creation

This is an important step that is often skipped. Researchers often run to publish before sharing findings with the researched communities. In addition, it is paramount and critical to involve primary stakeholders in co-creating recommendations. Validation and co-creation foster ownership, legitimacy, advocacy and accountability. It is imperative to get the intended beneficiaries to acknowledge that what the researchers have found and concluded is indeed true and has their backing. This does not only give legitimacy to the findings but when the affected speak, the world is forced to listen.

## Impact journey

**Figure 2:**
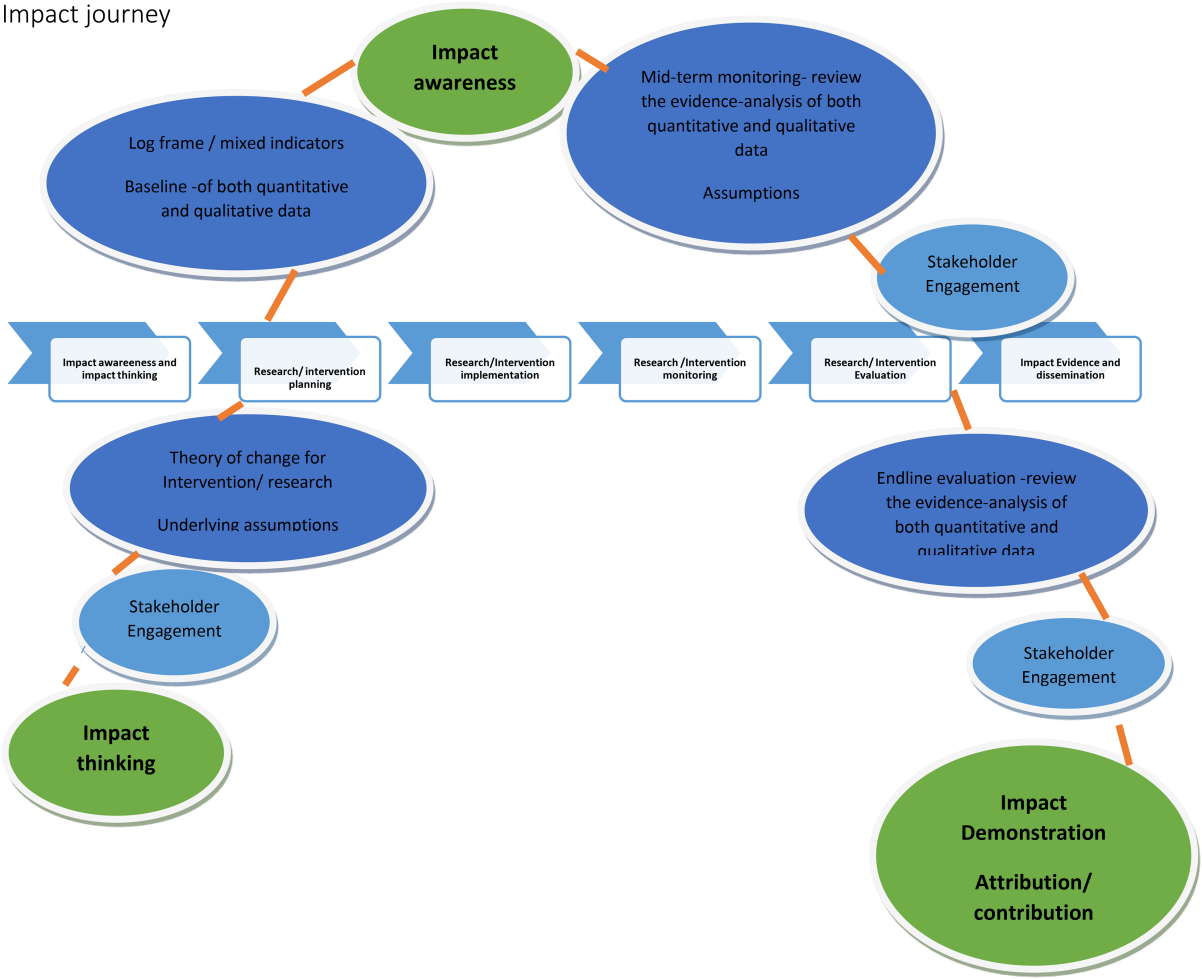
Impact journey

Impact evaluation can be viewed like a journey, with a starting point, mid-point and end point. The depiction is for simplification and we want to acknowledge that implementation is not linear and that in reality there are several feedback loops that are not evident in this diagram. Throughout, an assessment can be made if one is still on track. If off track, course correction is possible if the deviation is detected early enough (Figure 2).

### Touch points for impact

• Research intervention planning
• Research/Intervention implementation
• Research/Intervention monitoring
• Research Intervention evaluation

## Discussion

What is impact, who should assess impact, how, when and why are questions many researchers and implementers are grappling with as terms like human centered designs, co creation, participatory methods, evidence-based decision making, human rights-based approaches, value-based health care become main stream. The definition of impact has been given above. Who should measure impact? The answer is every-one entrusted with a research or intervention before anyone else. Any one entrusted with funding would like to know if they are utilizing the resources responsibly and if they are moving towards the set objectives (internal evaluation). An external evaluation can also be done by an impartial party, often at the behest of the funder. Financial, material and human resources are finite. How impact can be embedded at design stage has been described in detail in our attempt to demystify impact. The reason for measuring impact ranges from advocacy, accountability to decision making and resource allocation.

The question of attribution and contribution is inevitable. Contribution denotes that the influence is only one of many factors that contributed to a change in an outcome. Attribution denotes that the intervention is solely attributable to the observed change in the outcome. Ideally, an impact evaluation requires a counterfactual of what outcomes would have been in the absence of the intervention (4). Impact evaluation assesses the broader and long-term effects of a program answering the question, “What would have happened had the program been not implemented?” It compares outcomes of a program in one area to a counterfactual setting.

### Example

2 Nutrition programs, one providing peanut butter to be added in porridge at home and another providing lunch at school. School absenteeism can go down because the children got a good breakfast, porridge with peanut butter before school began or they stayed in school because of the school lunch provided. Can both programs claim attribution? Contribution is therefore more appropriate in instances of this nature.

The proposed impact evaluation framework is pragmatic. The essence is to demonstrate if the intervention has contributed to the outcome, demonstrated by the findings and supported by the program theory. If the evidence shown demonstrates attribution beyond any reasonable doubt, then impact can be claimed. In many settings, where multiple interventions are being rolled out simultaneously, it can be difficult to claim attribution. In such settings, impact thinking and the use of impact aware and complexity aware methods as described above can assist in demonstrating if the intervention program theory led to changes in the outcome. Demonstrating impact is not only restricted to experimental or quasi-experimental designs.

In summary, a pragmatic and practical approach to measuring impact is therefore needed.

• TOC, program theory causal pathway
• Stakeholder involvement, particularly primary stakeholders
• Baseline of outcome of interest
• Midline assessment of outcome of interest
• Endline assessment of outcome of interest
• Use of mixed methods indicators to assess the outcome of interest
• Validation/Co-creation

In the ideal world, the findings should be compared to a counterfactual. In our view, there will always be counterfactuals-those areas where the intervention was not rolled out, hence this should not be viewed as stumbling block

## Conclusion

There is recognition in prevention science to move from black box intervention approaches towards approaches that can elaborate on mechanisms through which activities lead to outcomes (6). A move away from the black box entails being explicit about how the desired goals have been achieved. It entails thinking behind why all programs should have ToCs, baseline measurements of outcome of interest etc. To that end we proposed the above impact evaluation framework to demystify impact evaluation by proposing the adoption of impact thinking and embedding of impact aware methods and indicators into programs research at design stage.

## Data Availability

Publicly available datasets were analyzed in this study. This data can be found here: N/A.
